# A Unique Triad: Ulcerative Colitis, Primary Sclerosing Cholangitis, and Autoimmune Hemolytic Anemia

**DOI:** 10.7759/cureus.2068

**Published:** 2018-01-15

**Authors:** Syeda Naqvi, Syed Askari Hasan, Sameen Khalid, Aamer Abbass, Melanie Albors-Mora

**Affiliations:** 1 Jinnah Postgraduate Medical Centre, Jinnah Sindh Medical University (SMC); 2 Internal Medicine Residency, Florida Hospital-Orlando; 3 Florida Hospital-Orlando

**Keywords:** ulcerative colitis, inflammatory bowel disease (ibd), primary sclerosing cholangitis

## Abstract

Ulcerative colitis is an autoimmune disorder leading to chronic intestinal inflammation. It can present with a wide range of associated extra-intestinal manifestations. We present a case of an 18-year-old man diagnosed with ulcerative colitis, autoimmune hemolytic anemia and primary sclerosing cholangitis during the same hospitalization. The unique triad of these diseases gives important clues to the immunological factors involved in the pathogenesis of these diseases.

## Introduction

 ​​​​Inflammatory bowel disease (IBD) is an autoimmune condition caused by a dysregulated immune response. IBD is composed of two major diseases: Crohn’s disease and ulcerative colitis (UC). UC is an inflammation of the mucosal layer of the colon and presents as bloody diarrhea, tenesmus, and abdominal pain due to colitis [1]. Systemic findings can be anemia due to iron deficiency, blood loss or in some instances, autoimmune hemolytic anemia (AIHA).

UC primarily involves the bowel with symptoms pertaining to colitis, but it is widely associated with extra-intestinal manifestations. Nearly 6% to 46% of patients have these extra-intestinal manifestations of UC, but the etiology remains unclear. The common associations are arthritis of the large joints, uveitis and episcleritis, erythema nodosum, and pyoderma gangrenosum, primary sclerosing cholangitis (PSC), fatty liver, venous thromboembolism, serositis, and parenchymal lung disease [2].

We present a unique triad of UC, PSC, and AIHA manifesting in a patient who presented to our emergency department.

## Case presentation

An 18-year-old man with no past medical history presented to the emergency department with concerns of bloody diarrhea, abdominal cramps, and nausea for the prior week. The patient was a visitor from the United Kingdom (UK). He denies any sick contacts, fever or rash. He was recently admitted to a hospital in the UK for similar complaints and was treated with two weeks of prednisone, but no diagnosis was established. He remained symptom-free for a month. He was a non-smoker, occasional alcohol user and denied illicit drug abuse. His family history was unremarkable.

On admission, he was afebrile, his blood pressure was 110/60 mm-Hg, and his pulse rate was 100 beats per minute. His abdominal examination revealed a non-distended abdomen that was diffusely tender on palpation.

Initial lab work showed a hemoglobin of 9.5 g/dL with a mean corpuscular volume 105 fl and reticulocyte count of 17%. His white blood cell count was 22x10^3 ^μL. His total bilirubin was 2.4 mg/dL with a direct bilirubin of 0.7 mg/dL, alanine aminotransferase of 133 μ/L, aspartate aminotransferase 83 μ/L and alkaline phosphatase 133 μ/L. His haptoglobin was 10 mg/dl.

**Figure 1:**
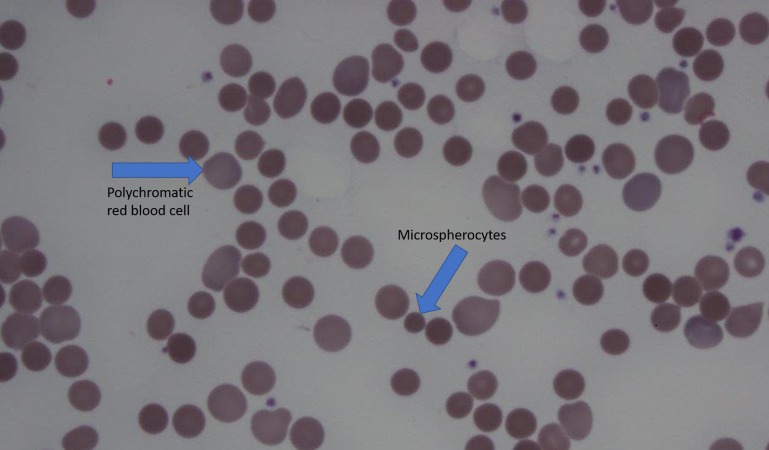
Peripheral Smear Arrows showing microspherocytes and polychromatic red blood cells

Further laboratory studies included hepatitis panel, the results of which were negative. His vitamin B-12 and folic acid levels were within normal limits. His peripheral smear showed microspherocytes and hemolysis by a warm antibody, noted in Figure 1.

**Figure 2:**
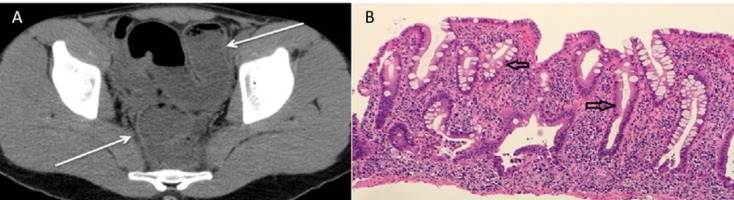
CT Scan (Figure A) and Intestinal Biopsy (Figure B) Figure A: Computed tomography (CT) scan demostrating mild wall thickness and featurelesss appearance of rectosigmoid colon Figure B: Descending colon at four times visulaization showing crypt distortion, crptitis and crypt abscess.

The results of the direct Coombs test were positive, and the results of the paroxysmal nocturnal hemoglobinuria testing were negative. The patient was diagnosed with AIHA and started on hydrocortisone (80 mg every eight hours). A complete stool infectious panel was ordered, and it was negative for *Shigella, Salmonella*, and *E. Coli*. Moreover, the result of his *Clostridium difficile *[PME4] toxin assay was negative and later confirmed by polymerase chain reaction. His computed tomography scan of the abdomen and pelvis showed mild wall thickening and featureless appearance of the rectosigmoid colon as noted in Figure 2. A colonoscopy showed mild to moderate pancolitis from the rectum to the ileocecal valve, and biopsies showed cryptitis, crypt abscesses, and crypt distortion consistent with UC, as noted in Figure 2.

**Figure 3:**
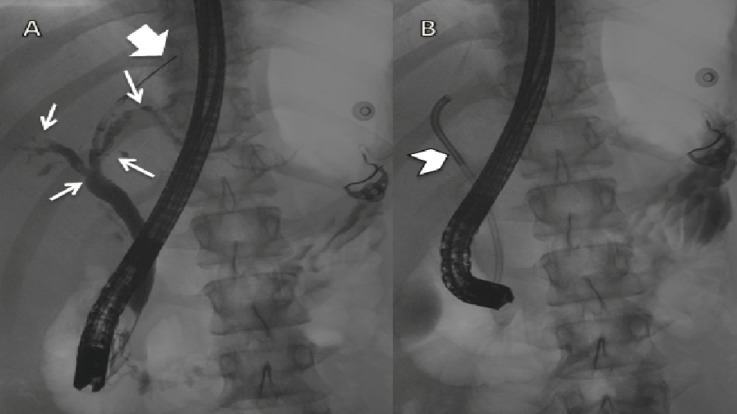
Magnetic Resonance Cholangiopancreatography Figure A: arrows pointing towards beaded appearance of intrahepatic ducts. Figure B: Endoscopic retrograde cholangiopancreatography (ERCP) image after biliary stent placement (arrowhead) with decompression of biliary tree.

The patient was started on a mesalamine 1.2-gram delayed-release tablet once daily. Magnetic resonance cholangiopancreatography (MRCP) showed cholangitis with the irregular beaded appearance of intrahepatic and extrahepatic bile ducts as noted in Figure 3.

The patient tested positive for anti-smooth muscle antibody and anti-neutrophil cytoplasmic antibody. An endoscopic retrograde cholangiopancreatogram (ERCP) was performed, and biopsies were taken from the ducts with chronic inflammation and no evidence of malignancy. A biopsy of the liver was performed to rule out autoimmune hepatitis and overlap syndrome. The patient improved clinically. His hemoglobin level remained stable. He was discharged to follow-up with a gastroenterologist in the UK.

## Discussion

UC is an autoimmune disorder involving the gastrointestinal tract, and common clinical presentations are bloody stools, abdominal pain, fever, and tenesmus. Acute complications can be bleeding, perforation, and fulminant colitis. A broad spectrum of extra-intestinal manifestations involving cutaneous, hepatobiliary, and ocular systems can occur in association with UC [2].

The probable mechanism of extra-intestinal manifestations is two-fold: antigen mimicry and genetic susceptibility. Factors like trauma or infection can expose the antigens in the blood resulting in autoantibodies. Colonic epithelial protein and human tropomyosin isoform 5 are antigens found recently in the intestinal mucosa. UC patients who display the HLA-B8, DR3 phenotype have a 10-fold higher risk of developing PSC. PSC should be suspected in a patient with deranged liver function tests (LFTs) and can be confirmed by MRCP and liver biopsy [3-4].

UC is associated with the development of primary sclerosing cholangitis in 3% to 5% of patients [4-5]. PSC is denoted by destructive inflammation of the intrahepatic and extrahepatic biliary ducts, clinically characterized by jaundice and confirmed by deranged LFTs, hepatomegaly, and imaging techniques like ultrasound and ERCP. For PSC, ursodeoxycholic acid has proven to be the only effective pharmacological treatment, and liver transplant is the only curative option [6]. However, the literature is full of a different range of treatment modalities tried on UC patients with AIHA. These treatment options include corticosteroids, mesalamine, azathioprine, cyclosporine, infliximab, stem cell transplant, colectomy, and splenectomy [7-8]. PSC is a precancerous condition with increased risk for cholangiocarcinoma and colorectal cancer, which indicates an intensive follow-up in these subgroups of patients.

Most common types of anemia associated with UC are an iron deficiency and anemia of certain chronic diseases. The presence of immune cytopenia is rare, but a few case reports have cited the occurrence of autoimmune hemolytic anemia and immune thrombotic thrombocytopenia with UC. In a multicentric trial, Uzzan, et al. described the incidence of autoimmune hemolytic anemia in UC as quite a bit higher compared to that of the general population [1]. AIHA is defined as a hemoglobin level below 10 g/dL with at least two features of hemolysis and a positive direct antiglobulin test. Our patient had multiple features including an increased reticulocyte count, low haptoglobin, and a peripheral smear showing micro spherocytes. The most accepted hypothesis of this phenomena is the cross-reactivity between colonic antigens and erythrocyte antigens.

AIHA is a condition in which there is a positive antibody against erythrocyte membrane [9]. AIHA with PSC is extremely rare. In a review of the literature, we noted one case reported in a 17-year-old female patient who presented with AIHA and PSC; the case described such a constellation in the absence of UC [9-10].

## Conclusions

This unique case of UC with primary sclerosing cholangitis and autoimmune hemolytic anemia in a young man highlights a rare association between these autoimmune conditions. The coexistence of UC with AIHA and PSC may also provide a better understanding of the complex interactions of the immune system in the pathogenesis of these diseases. Further research is needed to determine the genetics involved and treatment options for such occurrences. In UC patients, a vigorous workup is important for early detection of other autoimmune diseases to improve patient quality of life and to prevent associated mortality.
